# Perched Anteromedial Radial Head Dislocation

**DOI:** 10.1016/j.jhsg.2023.01.003

**Published:** 2023-02-09

**Authors:** Allison Rao, Edward Wu, Jonathan Braman, Alicia Harrison

**Affiliations:** ∗ Department of Orthopedic Surgery, University of Minnesota Medical School, Minneapolis, MN

**Keywords:** Closed reduction, Coronoid, Radial head, Radial head dislocation, Radius

## Abstract

Perched anteromedial radial head dislocation is a rare injury pattern that is yet to be reported in the literature. This article describes a case report of an isolated radial head dislocation that was perched on the coronoid process. The images in this study show this rare injury pattern, which did not include a fracture of the coronoid or true elbow dislocation. The patient was successfully treated with a closed reduction. The patient regained full ROM and function. Previously described literature has failed to report this injury pattern or successful closed treatment. The success of this case demonstrates the difficulty of closed reductions even under proper anesthesia and the importance of performing them in the setting where the surgeon has the option to convert to open reduction in unsuccessful cases.

Posttraumatic anterior radial head dislocation is an uncommon injury in the spectrum of adult elbow dislocations.^1^ When encountered, radial head dislocations typically occur in the setting of Monteggia fractures, Essex-Lopresti fractures, or in “terrible triad” injuries. We present a case report of a locked elbow due to an isolated radial head dislocation that was successfully treated with closed reduction under general anesthesia.

## Case Report

**Figure 1:**
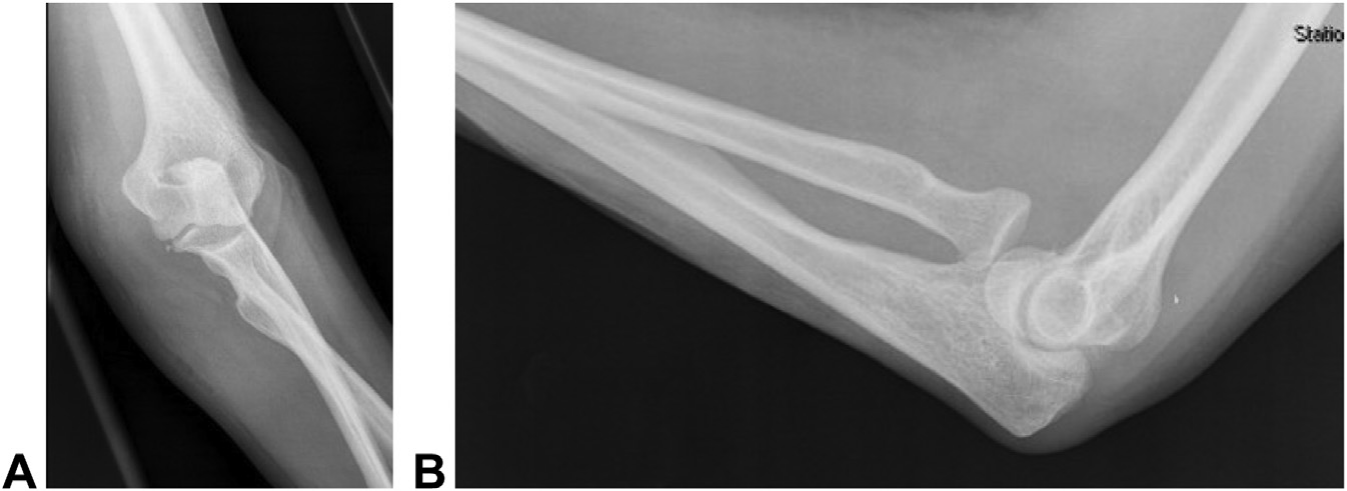
**A** Anteroposterior and **B** lateral radiographs at presentation demonstrate anteromedial dislocation of radial head without associated fractures.

Informed consent was obtained from the patient for publication of this case report and accompanying images. A 64-year-old right hand–dominant woman sustained an injury in the left upper extremity after tripping on a sidewalk. Physical examination in the ED showed a palpable fullness in the left antecubital fossa. Vascular and neurological examination demonstrated intact pulses and sensation proximal and distal to the elbow. She had no active or passive ROM at the elbow because of pain, and her forearm was locked in 0° of pronosupination. Radiographs showed anteromedial dislocation of the radial head with incarceration of the radial head on the anterior aspect of the radial notch of the ulna (Fig. 1).

**Figure 2:**
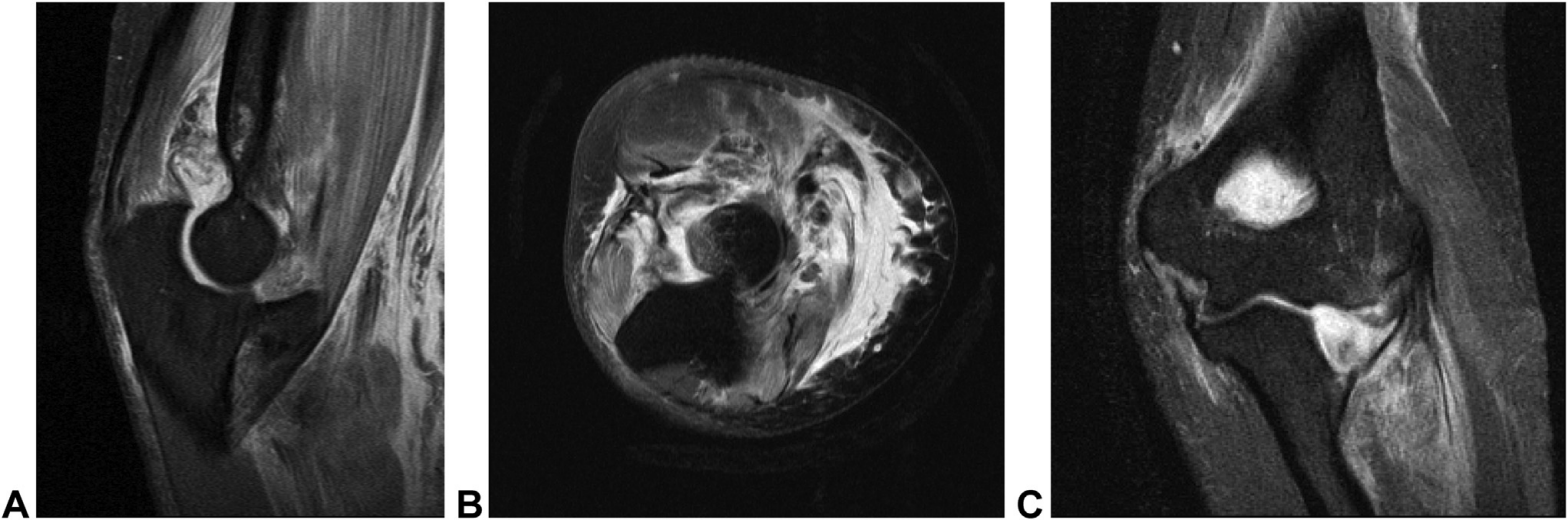
**A** Sagittal, **B** axial, and **C** coronal elbow MRI at presentation demonstrate anteromedial dislocation with posterior radial head impaction fracture.

The ED physician and hand surgeon attempted closed reduction under conscious sedation but were unsuccessful. Following the second attempt, the patient was sent for MRI (Fig. 2) of the left elbow and then placed in a posterior splint. The MRI showed an anteromedial dislocation of the radial head with a perched radial head impaction fracture. Soft tissue findings included a disruption of the anterior band of the radial collateral ligament, high-grade sprain of the lateral ulnar collateral ligament, and disruption of the annular ligament. No fracture of the coronoid was identified. Treatment options were discussed with the patient in the office, which included a repeat attempt at closed reduction under anesthesia and if unsuccessful, then, an open reduction with potential need for reconstruction or repair of the lateral collateral ligament. Risks discussed with the patient included recurrent instability, posttraumatic arthritis, stiffness, pain, and neurovascular injury.

**Figure 3:**
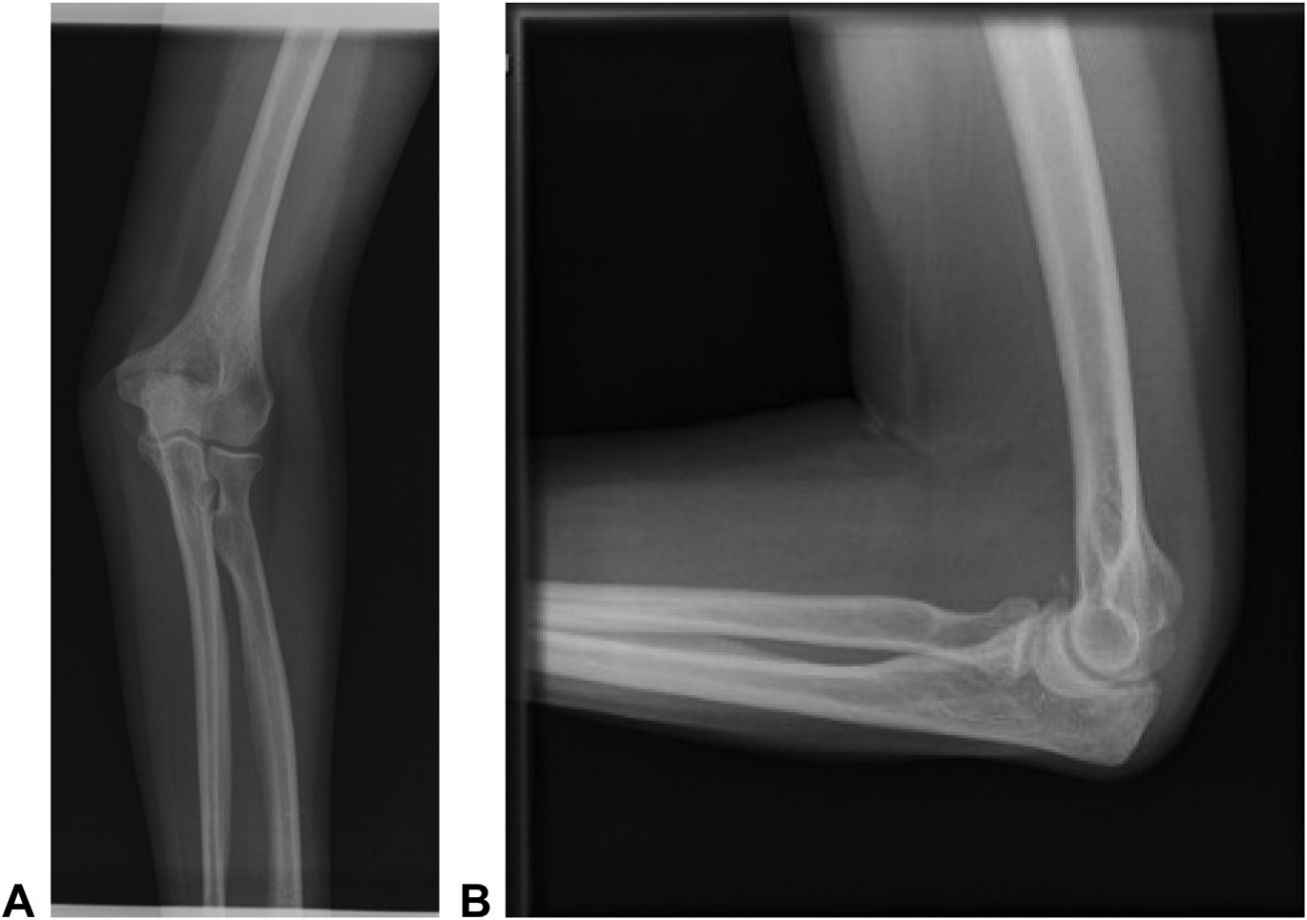
**A** Anteroposterior and **B** lateral views 1 year after closed reduction show normal radiocapitellar alignment.

In the operating room, examination under anesthesia demonstrated nearly full flexion and extension but locked pronosupination at 0°. The surgeon started with attempted closed reduction by applying a varus stress in full extension with gentle pronation, resulting in reduction. This maneuver involved recreation of the deformity and translation of the radial head laterally, followed by pronation for successful reduction. Postreduction clinical and fluoroscopic stress testing demonstrated no posterolateral rotatory instability with a negative pivot shift test. Upon pronation testing, however, the elbow redislocated at 45° of pronation with forearm flexion. Closed reduction was repeated, and the joint remained stable throughout a full ROM of flexion-extension and pronation/supination. The patient was placed in a splint with approximately 80° of flexion and maximum supination and was imaged to verify maintenance of reduction. The patient was asymptomatic with full ROM and stability on physical examination at 2-week, 6-month, and 1-year follow-ups after closed reduction. Radiographic imaging at each visit showed anatomic alignment at the radiocapitellar joint without fractures (Fig. 3). Imaging at 1 year showed resolution of the joint infusion and a small calcific density.

## Discussion

Isolated radial head dislocation without concurrent radial head or ulnar fractures is an uncommon presentation in adult patients. Of these dislocations, anterior and anteromedial dislocations are particularly rare with only a handful of published case reports in the literature.^2^ There is no consensus regarding the mechanism of injury resulting in anterior radial dislocation. Multiple mechanisms have been proposed across several case reports, which include direct trauma to a semiflexed elbow,^3^ hyperpronation of an extended arm,^4^ and hyperpronation with elbow flexion and varus strain.^5^

Currently, no classification system has been defined for isolated radial head dislocations, but injury patterns are commonly described by direction of displacement of the radial head. Most authors agree that the mechanism of injury requires hyperpronation or hypersupination, with hyperpronation resulting in anterior dislocation and hypersupination leading to posterior dislocation.^6^ Rupture and articular interposition of the annular ligament can cause the radial head to appear laterally displaced and has been implicated in irreducible dislocations.^2^^,^^7^^,^^8^ However, anteromedial displacement with the radial head incarcerated on the coronoid process as in our patient’s case has not been described. An elbow locked in pronosupination should raise suspicion for a bony block to reduction because recognizing this pattern is critical to successful closed treatment of this injury.

Although opinions regarding mechanisms of injury vary, there is general consensus that prompt reduction is critical for patient outcomes. Expeditious reduction benefits both patient and physician because prolonged time to treatment is associated with increasingly difficult reduction.^9^ Missed or improperly reduced radiocapitellar joints are prone to restriction of pronation/supination, posttraumatic arthritis, nerve injuries, and complications at the DRUJ.^2^

Closed reduction is first-line management but is often impeded by notable soft tissue damage and swelling.^7^ Radial head dislocations are associated with disruption of the annular ligament that can physically obstruct reduction, as well as varying degrees of lateral collateral ligament injury.^2^^,^^7^^,^^8^ Open reduction with annular and lateral collateral ligamentous repair or external fixation are used when closed reduction fails to restore joint stability.^10^

Our case describes an anteromedial radial head dislocation that perched on the ulna, resulting in a locked elbow to pronosupination with maintained passive flexion and extension. This interesting finding reinforces the fact that there was maintained stability in the ulnohumeral joint that is consistent with the intact lateral ulnar collateral ligament seen on MRI. Literature review reveals few adult cases of anterior radial head dislocations. Most published reports describe anterior dislocations from pronation of an extended elbow.^2^ Literature review revealed 2 cases of anterior radial head dislocation that were successfully treated with closed reduction.^2^^,^^4^ In contrast to these 2 cases, at presentation, our patient had a radial head impacted on the ulna resulting in locked pronosupination with preserved flexion and extension. Furthermore, our patient regained complete ROM following 2 weeks of immobilization, whereas the others lost ROM.^2^^,^^4^

In the event of an irreducible closed joint, 2 published cases report the efficacy of open reductions.^3^^,^^10^ In 1 case, closed reduction was impeded by a disrupted annular ligament. A successful outcome was achieved following open reduction with transcapitellar pinning.^3^ Another case report described a similarly incarcerated radial head with radial head dislocation but with concomitant proximal RU dislocation.^8^ The patient presented with a limited ROM in both flexion and pronation and was unable to achieve reduction with closed techniques. Open reduction revealed the radial head dislocated anteromedially and incarcerated on the medial aspect of the coronoid process with disruption of ulnar and radial collateral ligaments. At follow-up, both patients were asymptomatic and returned to their activities with small losses in ROM.^3^^,^^8^

Interestingly, in both cases of open reduction, the annular ligament was disrupted and in 1 report explicitly impeded reduction.^3^^,^^8^ Although our patient had displacement of the annular ligament into the joint space, closed reduction was successful. Furthermore, disruption of the ulnar collateral and radial collateral ligaments did not affect our patient’s stability or outcomes. These observations suggest that open annular and collateral ligament repairs are not necessary in the case of reducible, stable joints.

This case report supports 1 proposed mechanism of injury and highlights the success of closed reduction under anesthesia. Furthermore, it demonstrates the difficulty of reducing this uncommon injury without general anesthesia. Even with conscious sedation, 2 attempts were unsuccessful. Consequently, we recommend a high index of suspicion when reviewing injury films with this pattern of injury. This is particularly important in patients with absent pronosupination but with preserved flexion and extension. In this setting, we recommend performing a closed reduction under general anesthesia in the operating room so that conversion to an open procedure can be pursued if closed treatment is unsuccessful.
